# Mapping the interactome of overexpressed RAF kinase inhibitor protein in a gastric cancer cell line

**DOI:** 10.1186/1471-2407-13-536

**Published:** 2013-11-09

**Authors:** Huan Gu, Xianquan Zhan, Guiying Zhang, Lu Yan, William CS Cho, Maoyu Li, Ting Liu, Zhuchu Chen

**Affiliations:** 1 Department of Gastroenterology, Xiangya Hospital, Central South University, Changsha, Hunan 410008, China; 2 Key Laboratory of Cancer Proteomics of Chinese Ministry of Health, Xiangya Hospital, Central South University, Changsha, Hunan 410008, China; 3 Department of Clinical Oncology, Queen Elizabeth Hospital, Hong Kong, China

**Keywords:** Affinity chromatography, Co-immunoprecipitation, Fusion protein expression, Gastric cancer, RAF kinase inhibitor protein (RKIP), Tandem mass spectrometry

## Abstract

**Background:**

Gastric cancer (GC) is a threat to human health with increasing incidence and mortality worldwide. Down-regulation or absence of RAF kinase inhibitor protein (RKIP) was associated with the occurrence, differentiation, invasion, and metastasis of GC. This study aims to investigate the molecular mechanisms and biological functions of RKIP in the GC biology.

**Methods:**

The fusion expression plasmid pcDNA3.1-RKIP-3xFLAG was transfected into SGC7901 cells, the RKIP fusion proteins were purified with anti-flag M2 magnetic beads, and the RKIP-interacting proteins were identified with tandem mass spectrometry (MS/MS), and were analyzed with bioinformatics tools. Western blot and co-immunoprecipitation were used to confirm the interaction complex.

**Results:**

A total of 72 RKIP-interacting proteins were identified by MS/MS. Those proteins play roles in enzyme metabolism, molecular chaperoning, biological oxidation, cytoskeleton organization, signal transduction, and enzymolysis. Three RKIP-interaction protein network diagrams were constructed with Michigan Molecular Interactions, functional linage network, and Predictome analysis to address the molecular pathways of the functional activity of RKIP. The MS/MS-characterized components of the existing interaction complex (RKIP, HSP90, 14-3-3ϵ, and keratin 8) were confirmed by Western blot analysis and co-immunoprecipitation.

**Conclusion:**

This study is the first discovery of the interaction of RKIP with HSP90, 14-3-3, and keratin. The present data would provide insight into the molecular mechanisms of how RKIP inhibits the occurrence and development of GC.

## Background

The incidence of gastric cancer (GC) ranks fourth among cancers in the world, and its incidence and mortality rank second among malignant tumors of the digestive tract [1,2]. A total of 750,000 patients die from GC each year in the world, including 160,000 patients in China [1]. The pathogenesis of GC still remains unclear. Early gastric diagnosis, the prediction of relapse and metastasis, and prognosis assessment are of importance for GC prevention. Therefore, searching for new tumor markers and gene therapy targets is of high priority.

Our previous laser-capture microdissection (LCM)-based quantitative proteomics studies found that RAF kinase inhibitor protein (RKIP) was significantly down-regulated in the GC tissues compared with the normal gastric mucosa tissues [3,4]. RKIP is a small (21 kDa), highly conserved cytoplasmic protein, and is a member of the phosphate ester acyl diethanolamine-binding protein family that participates in lipid metabolism and phospholipid membrane formation [5]. RKIP is extensively expressed in a variety of tissues and different cell types with multiple physiological and pathological functions [5]. The abnormal expression of RKIP plays an important role in the growth and differentiation process of GC [6] with evidences that a positive correlation between RKIP expression and the degree of differentiation of the GC tissue and a negative correlation between RKIP expression and tumor infiltration depth, TNM staging, and lymph node metastasis were found by immunohistochemistry and Western blot analyses [3,4].

Moreover, many studies have demonstrated a negative correlation between RKIP protein and the metastatic ability of malignant tumors such as prostate cancer, breast cancer, colon cancer, melanoma, and adenocarcinoma, as well as the potential anti-cancer activity of RKIP protein, which would be a novel therapeutic target for cancer [7-14]. Besides, RKIP protein can promote the apoptosis of tumor cells. It was reported that after treatment with chemotherapy drugs, breast cancer and prostate cancer cells can induce RKIP protein expression and cell apoptosis [15]. The decreased expression of RKIP in breast cancer cells and prostate cancer cells inhibits chemotherapy-induced apoptosis, whereas the restoration of RKIP expression can increase the sensitivity of the tumor cells to chemotherapy drugs [15]. The loss of abnormally expressed RKIP functions increases the resistance of cancer cells to chemotherapy, which causes the growth of the tumor cells [12]. Therefore, the down-regulation or absence of RKIP expression plays an important role in the development of tumors.

**Figure 1:**
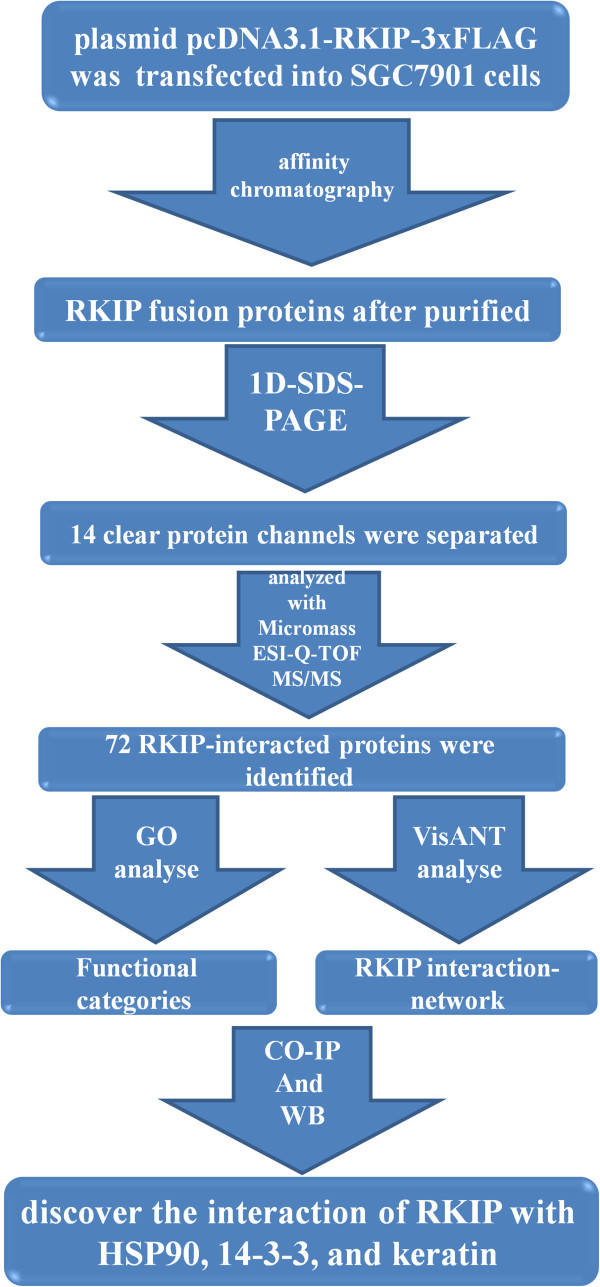
Overall scheme of the experiments used to identify and analyze the proteins that interact with RKIP in the GC biology.

Study found that RKIP interacts with a number of different proteins and regulates multiple signaling pathways [16,17]. However, the molecular mechanisms, biological functions, and the interacted proteins of RKIP inhibiting the occurrence and metastasis of GC remain unclear. Targeted proteomics is an effective approach for investigating the molecular mechanisms and biological functions of a given protein that is associated with a disease. Meanwhile, the 3xFLAG system coupled with highly specific and sensitive anti-FLAG antibodies is an effective and optimized approach for detection of fusion protein and its interacting proteins [18-20], and has successfully used to study the PPP1CC2-interacting proteins in transgenic mouse embryonic stem cells [21], NLRC4 phosphorylation [22], BAP1-interacting proteins [23], HMGB1-interacting proteins [24], FAT10-interacting proteins [25], and CyaA-interacting proteins [26]. In this study, the targeted proteomic strategy combined 3xFLAG-based affinity purification and tandem mass spectrometry to isolate and identify the RKIP-related proteins from the RKIP-overexpressed SGC7901 GC cell line (Figure 1). This study would provide insight into the molecular mechanisms of how RKIP inhibits the occurrence and development of GC.

## Methods

### Instruments and software

IPGphor, Ettan DALT II System, Image-Scanner (maximum resolution 9,600 × 9,600 dpi; Amersham Biosciences, Stockholm, Sweden), electrospray ionization-quadrupole-time of flight (ESI-Q-TOF) Mass Spectrometer (Micromass, Manchester, UK), PDQuest system (Bio-Rad laboratories, Hercules, CA), Mascot Distiller and Mascot Database Search engine, and Statistical Package For Social Science (SPSS for windows, Version 17.0, Chicago, IL, USA) were used.

### Cell lines and plasmid

Human gastric carcinoma cells (SGC7901) were purchased from the Laboratory for Cancer Research (Central South University, China). SGC7901 cells were cultured with RPMI1640 medium containing 10% fetal calf serum (Gibco BRL, Grand Island, NY). The pCDNA3.1-RKIP-3xFLAG plasmid, pcDNA3.1-3xFLAG plasmid, and pcDNA3.1-RKIP plasmid were purchased from Yingrun Biotechnology Co., Ltd. A total of four experimental groups were set up: SGC7901 cells tranfected with pcDNA3.1-RKIP-3xFLAG plasmid (RKIP-3xFLAG group), SGC7901 cells tranfected with pcDNA3.1-3xFLAG plasmid (3xFLAG group), SGC7901 cells tranfected with pcDNA3.1-RKIP plasmid (RKIP group), and SGC7901 cells (Blank group).

### Transfection

SGC7901 cells were recovered, cultured for logarithmic cell growth, then before transfection SGC7901 cells were plated into 15-cm^2^ petri dishes. The cells were used for transfection when the cell reached 90% confluency and were assigned to either the RKIP-3xFLAG group (fusion carrier group), the 3XFLAG group (blank carrier group), RKIP group (which was used to validate whether 3xFLAG affects the expression of RKIP), or the blank group. Transfection was conducted according to the LipofectamineTM2000 instructions for liposome transfection.

### Western blot analysis of RKIP and fusion proteins

The expressions of RKIP proteins and RKIP-3xFLAG fusion proteins were detected by Western blot analysis after transfection. The procedure was performed as follows: the cells were collected from the flasks, washed three times with cold PBS, and lysed (4°C, 30 min) in a lysis buffer. The protein concentration was determined with a protein assay kit (Bio-Rad laboratories). Protein extracts (about 20 mg) were subjected to SDS–PAGE with a 10% acrylamide gel. The gel-separated proteins were transferred to PVDF membranes (Millipore Corporation, Bedford, MA), incubated with primary antibodies, including anti-RKIP, anti-Flag, and anti-β-actin antibodies (Santa Cruz biotechnology, Santa Cruz, CA), and probed with secondary antibodies. The PVDF membranes with protein-antibody complexes were washed tree times with TBST buffer. The proteins on the PVDF membranes were visualized with the enhanced chemiluminescence (ECL) detection system. Western blot analysis was repeated at least three times.

### Purification of RKIP fusion proteins

The proteins from the RKIP-3xFLAG group, 3xFLAG group, and blank group were purified according to the FLAG M2 magnetic beads manual procedures of protein purification (each experimental step was conducted at 2–8°C), respectively. Briefly, an adequate amount of affinity gel in a clean centrifuge tube was centrifuged (5,878 x g, 30 sec) and was allowed to precipitate (approximately 2 min). The supernatant was discarded and the precipitate was washed twice with TBS solution that was equivalent to 20-fold volumes of the magnetic bead solution. The supernatant was discarded, and the pellet was washed with 0.1 M glycine (pH 3.5) HCl (20 min). The protein samples and affinity gel were mixed and incubated (overnight, 4°C). The incubated mixture was centrifuged (4°C, 5,878 x g, 30 sec), and the supernatant was carefully removed. The pellet was treated with a pre-chilled solution. The proteins from each group were denatured in a boiling water bath (3 min), centrifuged (2,296 x g, 30 sec), and stored at low temperature (-80°C) for further analyses.

### MS/MS-identification of proteins

After 1D SDS-PAGE separation of the purified proteins from three groups (RKIP-3xFLAG, 3xFLAG, and Blank), respectively,the proteins that were contained in the gel bands were digested with trypsin, and the tryptic peptide mixture was analyzed with Micromass ESI-Q-TOF MS/MS. The tryptic peptide samples were loaded onto a pre-column (320 μm × 50 mm, 5 μm C18 silica beads; Waters) to be concentrated and quickly desalted (30 l/min flow-rate) through a Waters CapLC autosampler. The concentrated and desalted tryptic peptides were online eluted to the reversed-phase column (75 μm × 150 mm, 5 mm, 100 Å, LC Packing) at a flow-rate of 200 nl/min. MS/MS spectra were acquired in a data-dependent mode in which up to four precursor ions above an intensity threshold of seven counts/second (cps) from each survey “scan” were selected for MS/MS analysis. The nanospray parameters were the following: a 3,000 V capillary voltage, a 45 V cone voltage, an 80°C source temperature, and a 15-psi collision gas back pressure. The MS/MS data were used to search the identified proteins against the protein database with the Mascot search engine. The search parameters used were the following: homo sapiens as the current species, a mass tolerance of +0.5 Da, an MS/MS tolerance of +0.3 Da, up to 1 missed cleavage site, fixed carboxymethyl (cysteine) modification, variable oxidation (methylation) modification, the Micromass PKL format, and the ESI Q-TOF instrument. The proteins identified from the blank-carrier control group and from the blank control group were regarded as non-specific proteins, and were removed from the protein list identified from the pcDNA3.1-RKIP-3xFLAG test group to rule out the non-specifically binding proteins of RKIP.

### Co-immunoprecipitation

A volume (1 ml) of extraction buffer that contained 1.5 mg of protein from cells was mixed with 5 μl of non-immune rabbit serum and 50 μl of protein G-sepharose 4B beads was oscillated (40°C, 2 h) and centrifuged (2,296 x g, 3 min) to eliminate the nonspecific binding of proteins. After centrifugation, the supernatant was retained and mixed with 20 μg of RKIP antibodies and 50 μL of protein G-sepharose 4B beads (40°C, oscillation, 3 h). The mixture was centrifuged (2,296 x g, 3 min). The beads were retained and washed three times with TBST buffer. Non-immune IgY antibodies, instead of RKIP antibodies, were used as controls.

### Construction of the RKIP-interaction protein network diagrams

VisANT 3.8.6 software (http://visant.bu.edu/) was used to analyze and construct the RKIP-interaction protein network diagrams. VisANT is an interactive software platform that is to visualize, mine, analyze, and model biological networks. When VisANT was used in this study, homo sapiens was selected for the current species parameter, and the Uniprot IDs of RKIP and of the 72 identified proteins were entered into the search box. Three databases, including Michigan Molecular Interactions (MiMI), functional linage network, and Predictome, were chosen to acquire the interaction network diagrams. The MiMI tool was provided by the National Institute of Health’s National Center for Integrative Biomedical Informatics. The MiMI tool provides access to the knowledge and data that have been merged and integrated from numerous protein-interaction databases, and it augments the information from many other biological sources. The Predictome database is based on the implementation of published computational methods and publicly available data and can precisely predict the connections between proteins; the associations are created using a variety of techniques, both experimental (yeast two-hybrid, immuno-coprecipitation, correlated expression) and computational (gene fusion, chromosomal proximity, gene co-evolution).

For the constructed protein network diagrams, each protein was located on a different level based on the interaction between that protein and RKIP. The 1st level neighbors were the directly interacting proteins, the 2nd level neighbors are the secondary interacting proteins, and the 3rd level neighbors were the tertiary interacting proteins. The 1st and 2nd level neighbors in the RKIP-interaction protein networks were considered to be the closely interacting proteins of RKIP because the interactions of RKIP with the 1st and 2nd level neighbors were much closer than those with the other level neighbors.

### Validation of RKIP-related proteins

Western blot analysis and co-immunoprecipitation were used to validate the interactions of HSP90, 14-3-3ϵ, and Keratin 8 with RKIP. The total protein from SGC7901 cells was precipitated in an appropriate lysis buffer containing RKIP antibody. The immunoprecipitated proteins were further analyzed by SDS-PAGE. Western blot analysis was used to detect HSP90, 14-3-3ϵ, and keratin 8 with their corresponding antibodies in order to study the target proteins’ interactions with RKIP. Non-immune IgY antibodies replaced the RKIP antibodies as negative controls (3×).

### Statistical analysis

All experiment data were expressed as mean ± SE and analyzed with Student’s *t*-test with a statistical significance level of p < 0.05.

## Results

### Expression of RKIP protein in transfected cells

**Figure 2:**
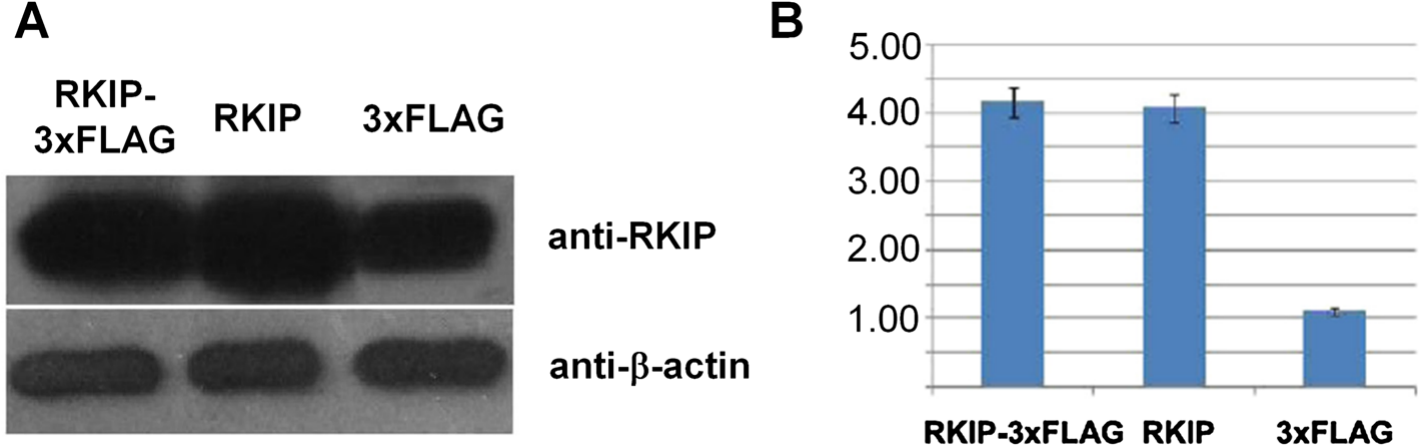
**The expression levels of RKIP in RKIP-3xFLAG, RKIP, and 3xFLAG groups. A**. Western blot image. **B**. The intensity of the bands in the Western blot image (p < 0.05). The 3xFLAG did not affect the expression of RKIP.

The expression level of RKIP protein in transfected cells was determined by Western blot analysis. The intensity of the Western blot images was analyzed with IPP 6.0 software and represented the relative amount of protein expression. The Western blot analysis shows that the RKIP expression levels of the RKIP-3xFLAG group and of the RKIP group were significantly higher than those of the 3xFLAG group (p < 0.05; Figure 2).

### Purification of RKIP fusion proteins

**Figure 3:**
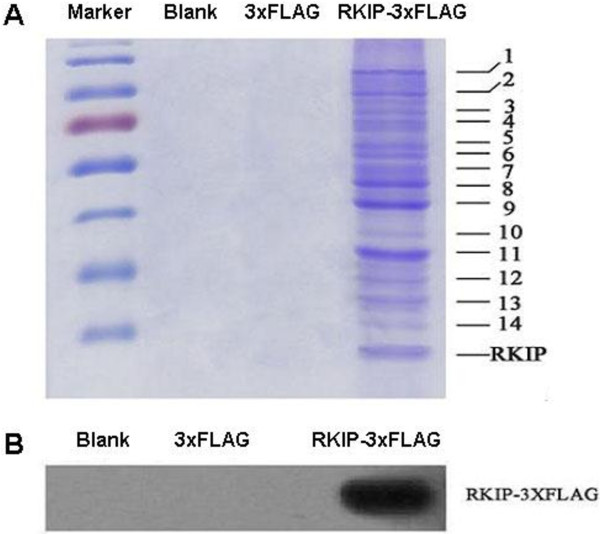
**Separation of RKIP-interacting proteins with anti-flag M2 magnetic beads and 1D SDS-PAGE. A**. After the affinity magnetic bead based purification, with anti-flag M2 magnetic beads, of total protein from the cells, the protein sample was pre-separated by 1D-SDS-PAGE using a 10% acrylamide gel. The clear channel proteins (n = 14) were separated in the experimental group. **B**. Western blot analysis to test RKIP-3xFLAG expression in different groups of affinity purified cells. After the affinity magnetic bead based purification, with anti-flag M2 magnetic beads, of total protein from the cells, RKIP-3xFLAG expression was detected in only the RKIP-3xFLAG group, whereas no RKIP-3xFLAG expression was detected in the 3xFLAG and blank groups.

After the affinity-magnetic bead purification, with anti-flag M2 magnetic beads, of the total protein from the cells, most of the protein sample was pre-separated by 1D-SDS-PAGE using a 10% acrylamide gel. The experiment was repeated three times with the same test conditions and parameter settings, and then the gel images were obtained with clear backgrounds, high resolution, and good reproducibility. A total of 14 RKIP interacting protein bands were identified (Figure 3A).

Western blot analysis was used to test the remaining protein samples. The results demonstrated that no cross-contamination between the experimental and control groups occurred; three reproducible tests confirmed the reliability (Figure 3B).

### Identification of RKIP-related proteins

**Figure 4:**
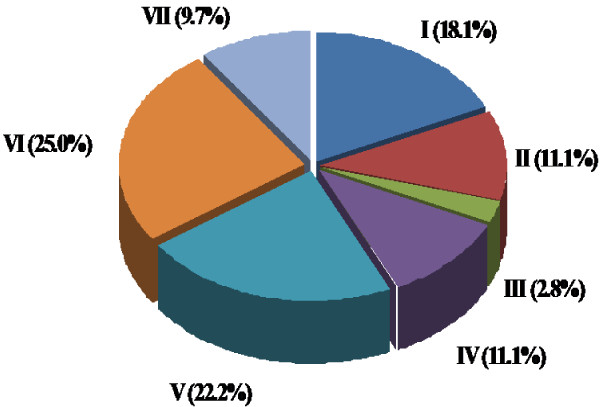
**Functional categories of RKIP-interacting proteins (n = 72).** I. Metabolic enzymes (n = 13; 18%); II. Molecular chaperones (n = 8; 11%); III. Biological oxidation-related proteins (n = 2; 3%); IV. Signal transduction-related proteins (n = 8; 11%); V. Cytoskeleton-related proteins (n = 16; 22%); VI. Protease-related proteins (n = 18; 25%); and VII. Others (n = 7; 10%).

The RKIP-related proteins were separated with 1D-SDS-PAGE and visualized with Coomassie brilliant blue R-250. The proteins in the gel-bands were subjected to in-gel trypsin digestion. The tryptic peptides were analyzed with ESI-Q-TOF MS/MS. The obtained MS/MS data were used to identify the proteins with Mascot software to search the Swiss-Prot or the NCBInr database; this experiment was repeated three times. The proteins that only appeared in the RKIP-3xFLAG group were chosen to effectively rule out the non-specific proteins. However, no proteins were identified by MS/MS in the 3xFLAG and blank groups, which is consistent with the results of gel-image without any protein band in the Figure 3. This confirmed that the identified proteins from the pcDNA3.1-RKIP-3xFLAG group were the RKIP-interacting proteins. A total of 72 proteins were MS/MS-identified (Table 1; Additional file 1: Table S1). The identified proteins belong to different functional categories (Figure 4; Table 1), including metabolic enzymes (n = 13; 18%), molecular chaperones (n = 8; 11%), biological oxidation-related proteins (n = 2; 3%), signal transduction-related proteins (n = 8; 11%), cytoskeleton-related proteins (n = 16; 22%), protease-related proteins (n = 18; 25%), and others (n = 7; 10%). The same proteins (14-3-3, keratin, filamin, tubulin, GSTP1, HSP90, DHX9, actin, and vimentin) were consistently identified in three repeated experiments.

**Table 1:** RKIP-related proteins identified with ESI-Q-TOF MS/MS

**UniProt ID**	**Protein name**	**Protein mass (Da)**	**Score**	**Matching peptides**	**Protein function**
*I. Metabolic enzymes*					
P53618	Coatomer subunit beta	108,241	89	3	Mediate biosynthetic protein transport
P14618	Pyruvate kinase isozymes M1/M2, isoform M2	58,470	604	40	Glycolytic enzyme
P00966	Argininosuccinate synthase	46,791	142	6	ATP binding
P00558	Phosphoglycerate kinase 1	44,992	102	3	Glycolytic enzyme
P08195	4F2 cell-surface antigen heavy chain, isoform 2	68,180	56	2	Function of light chain amino-acid transporters
P06733	Alpha-enolase	47,481	517	25	Multifunctional enzyme
P04406	Glyceraldehyde-3-phosphate dehydrogenase	36,201	769	80	Glyceraldehyde-3-phosphate dehydrogenase and nitrosylase activities
P07195	L-lactate dehydrogenase B chain	36,900	74	5	L-lactate dehydrogenase activity
P00338	L-lactate dehydrogenase A chain, isoform 1	36,950	43	4	L-lactate dehydrogenase activity
P40926	Malate dehydrogenase, mitochondrial	35,937	49	3	L-malate dehydrogenase activity
P46940	Ras GTPase-activating-like protein IQGAP1	189,761	55	3	Promote neurite outgrowth
P68104	Elongation factor 1-alpha 1	50,451	190	11	Protein biosynthesis
P02786	Transferrin receptor protein 1	85,274	74	3	Molecular transducer activity
*II. Molecular chaperone*					
P14625	Endoplasmin	92,696	192	18	Molecular chaperone ATPase activity
P07900	Heat shock protein HSP 90-alpha, isoform 2	85,006	280	24	Stress related, chaperone
P08238	Heat shock protein HSP 90-beta	83,554	276	25	Stress related, chaperone
P11142	Heat shock cognate 71 kDa protein, isoform 1	71,082	398	16	Stress related, chaperone
P38646	Stress-70 protein, mitochondrial	73,920	253	7	Related with cellular proliferation and aging
P10809	60 kDa heat shock protein, mitochondrial	61,187	723	22	Stress related, chaperone
P53992	Protein transport protein Sec24C	119,789	61	5	Component of the COPII coat
P50454	Serpin H1	46,525	133	3	Chaperone in the biosynthetic pathway of collagen
*III. Biological oxidation-related proteins*					
O00299	Chloride intracellular channel protein 1	27,248	78	3	Voltage-gated chloride channel activity, redox-regulated
Q06830	Peroxiredoxin-1	22,324	51	7	Redox regulation
*IV. Signal transduction*					
O43390	Heterogeneous nuclear ribonucleoprotein R, isoform 1	71,184	63	2	mRNA processing, ribonucleoprotein
P52597	Heterogeneous nuclear ribonucleoprotein F	45,985	110	2	mRNA processing, ribonucleoprotein
P04083	Annexin A1	38,918	454	24	Signal transduction, calcium ion binding
P12429	Annexin A3	36,524	53	4	Signal transduction, calcium ion binding
P08758	Annexin A5	35,971	50	10	Signal transduction, calcium ion binding
P62258	14-3-3 protein epsilon	29,326	136	6	Anti-apoptosis, signal transduction, transcription factor binding
P23396	40S ribosomal protein S3	26,842	122	9	NF-KappaB binding
Q99623	Prohibitin-2	33,276	38	5	Mediate transcriptional repression
*V. Cytoskeleton-related proteins*					
O75369	Filamin-B, isoform 1	280,157	91	1	Connects cell membrane constituents to the actin cytoskeleton
P21333	Filamin-A, isoform 2	283,301	91	3	Actin binding
P18206	Vinculin, isoform 1	124,292	668	30	Actin filament-binding protein
P04264	Keratin, type II cytoskeletal 1	66,170	89	4	Cytoskeleton of Intermediate filaments
P05787	Keratin, type II cytoskeletal 8	53,671	156	11	Cytoskeleton of Intermediate filaments
Q04695	Keratin, type I cytoskeletal 17	48,361	221	10	Cytoskeleton of Intermediate filaments
P05783	Keratin, type I cytoskeletal 18	48,029	547	35	Cytoskeleton of Intermediate filaments
Q15149	Plectin-1, isoform 1	533,462	73	11	Actin binding
P35579	Myosin-9, isoform 1	227,646	62	10	Play a role in cytokinesis, secretion
P68371	Tubulin beta-2C chain	50,255	245	19	The major constituent of microtubules
P07437	Tubulin beta chain	50,095	309	25	The major constituent of microtubules
P60709	Actin, cytoplasmic 1	42,058	617	36	Involved in various types of cell motility
P52907	F-actin-capping protein subunit alpha-1	33,073	61	3	Actin binding
P04075	Fructose-bisphosphate aldolase A	39,851	141	7	Actin binding
P08670	Vimentin	53,619	574	17	Cell motility, structural constituent of cytoskeleton
Q9NYL9	Tropomodulin-3	39,741	85	9	Actin binding
*VI. Protease-related proteins*					
P53396	ATP-citrate synthase	121,674	225	7	Enzyme regulator activity
P55072	Transitional endoplasmic reticulum ATPase	89,950	42	3	Necessary for the formation of transitional endoplasmic reticulum
Q08J23	tRNA (cytosine-5-)-methyl transferase NSUN2	87,214	78	2	RNA methyltransferase
P06576	ATP synthase subunit alpha, mitochondrial	59,828	158	9	Mitochondrial membrane ATP synthase
P60842	Eukaryotic initiation factor 4A-I	46,357	169	7	ATP-dependent RNA helicase
P55786	Puromycin-sensitive aminopeptidase	103,895	117	3	Involved in proteolytic events essential for cell growth and viability
P50395	Rab GDP dissociation inhibitor beta	51,095	78	3	Rad GDP-dissociation inhibitor activity
P42330	Aldo-keto reductase family 1 member C3	37,229	35	2	Aldo-keto reductase activity
P63244	Guanine nucleotide-binding protein subunit beta-2-like 1	35,511	112	3	Up-regulation of the activity of kinases
P61289	Proteasome activator complex subunit 3, isoform 1	29,602	77	2	Endopeptidase activator activity
P09211	Glutathione S-transferase P	23,569	56	30	Glutathione transferase activity
P13639	Elongation factor 2	96,246	240	9	Catalyzes the GTP-dependent ribosomal translocation
Q15029	116 kDa U5 small nuclear ribonucleoprotein component	110,336	130	6	Component of the U5 snRNP complex
P10696	Alkaline phosphatase, placental-like	57,626	140	8	Alkaline phosphatase activity
Q99714	3-hydroxyacyl-CoA dehydrogenase type-2, isoform 1	27,134	108	3	Mitochondrial tRNA maturation
Q08211	ATP-dependent RNA helicase A	142,181	264	12	Transcriptional activator
P41252	Isoleucyl-tRNA synthetase, cytoplasmic	145,718	94	9	Isoleucine-tRNA ligase activity
P26640	Valyl-tRNA synthetase	141,642	78	4	ATP binding
*VII. Others*					
Q13263	Transcription intermediary factor 1-beta, isoform 1	90,261	59	3	Nuclear corepressor for KRAB domain-containing zinc finger proteins
Q14974	Importin subunit beta-1	98,420	84	2	Protein domain specific binding
P26641	Elongation factor 1-gamma	50,429	65	2	Unknown
P21796	Voltage-dependent anion-selective channel protein 1	30,868	78	3	Voltage-gated anion channel activity
P61026	Ras-related protein Rab-1A, isoform 1	22,755	45	1	Involved in vesicular trafficking and neurotransmitter release.
Q00610	Clathrin heavy chain 1, isoform 1	193,620	36	3	The polyhedral coat of coated pits and vesicles
Q14683	Structural maintenance of chromosomes protein 1A	143,771	221	6	Involved in chromosome cohesion

### RKIP-interaction protein network diagrams and validation of the RKIP-protein complex

**Figure 5:**
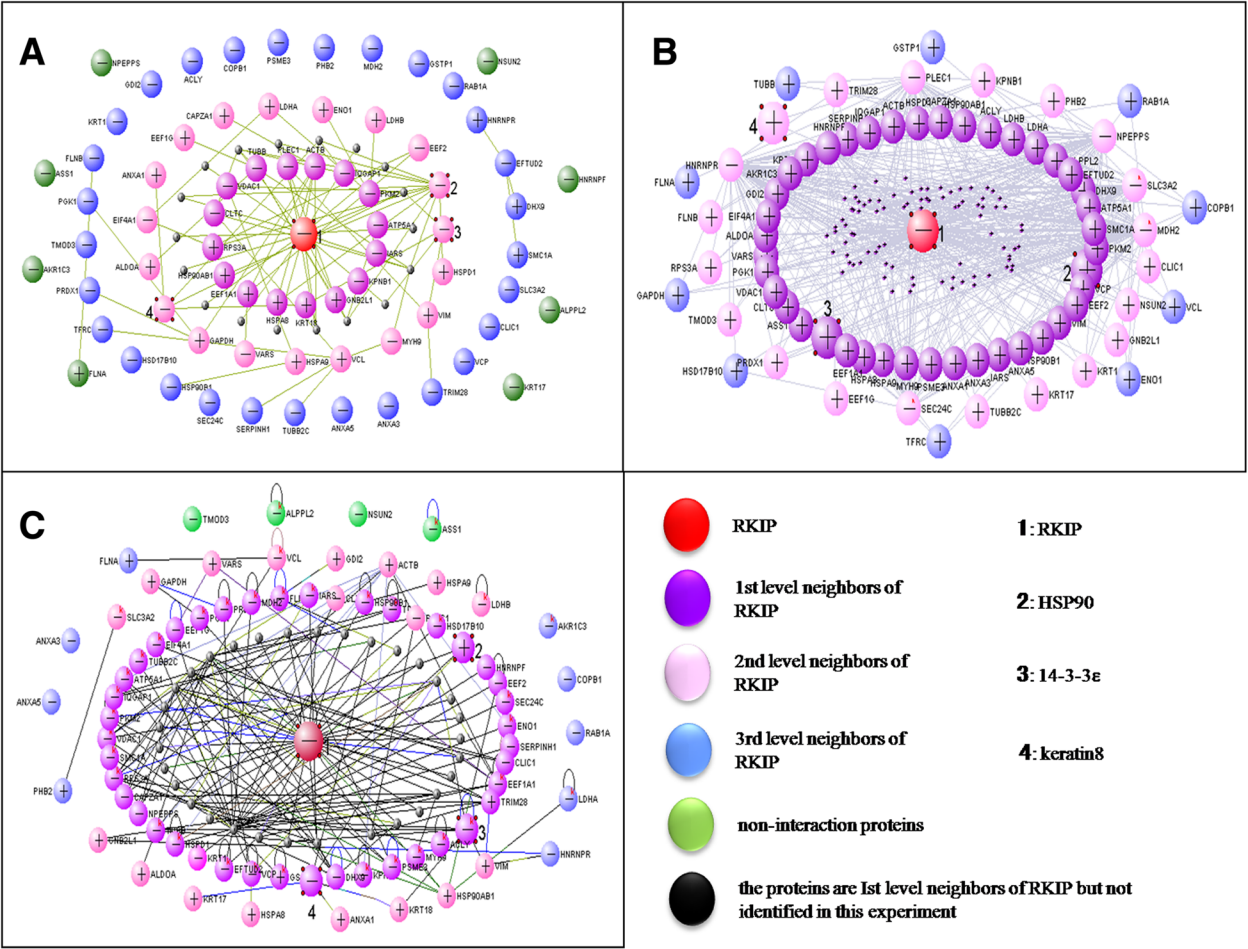
**The RKIP-interaction protein network diagrams.** Each protein network diagram was derived from the database retrieval with MiMI **(A)**, the functional linage network **(B)**, and Predictome **(C)**, respectively.

VisANT software with three functional models (MiMI, functional linkage network, and Predictome) was used to search for interactions between RKIP and the 72 identified proteins, to construct the RKIP interaction network diagram, and to define the levels of interaction between the identified proteins and RKIP (Figure 5).

In the protein network diagram that was derived from the database retrieval with MiMI, among the 72 RKIP-related proteins, 16 proteins were classified as the 1st level neighbors of RKIP, 19 proteins were classified as the 2nd level neighbors of RKIP, 29 proteins were classified as the 3rd level neighbors of RKIP, and 8 proteins were found to not interact with RKIP (Figure 5A, Table 2).

**Table 2:** **The levels of interaction between the identified proteins and RKIP that located in the RKIP-interaction protein network diagrams (Figure**5**), analyzed with the VisANT 3.8.6 protein-network analysis package**

**UniProt ID**	**Protein name**	**MiMI**	**Functional linage network**	**Predictome**
P46940	Ras GTPase-activating-like protein IQGAP1	1^st^	1^st^	1^st^
P08238*	**Heat shock protein HSP 90-beta**	**2**^**nd**^	**1**^**st**^	**1**^**st**^
Q14974	Importin subunit beta-1	1^st^	2^nd^	1^st^
P11142	Heat shock cognate 71 kDa protein	1^st^	1^st^	2^nd^
P14618	Pyruvate kinase isozymes M1/M2	1^st^	1^st^	1^st^
P06576	ATP synthase subunit alpha, mitochondrial	1^st^	1^st^	1^st^
P07437	Tubulin beta chain	1^st^	3^rd^	1^st^
P68104	Elongation factor 1-alpha 1	1^st^	1^st^	1^st^
P60709	Actin, cytoplasmic 1	1^st^	1^st^	2^nd^
P05783	Keratin, type I cytoskeletal 18	1^st^	2^nd^	2^nd^
Q15149	Plectin	1^st^	2^nd^	1^st^
P21796	Voltage-dependent anion-selective channel protein 1	1^st^	1^st^	1^st^
P23396	40S ribosomal protein S3	1^st^	2^nd^	1^st^
P63244	Guanine nucleotide-binding protein subunit beta-2-like 1	1^st^	2^nd^	2^nd^
Q00610	Clathrin heavy chain 1	1^st^	1^st^	1^st^
P41252	Isoleucyl-tRNA synthetase, cytoplasmic	1^st^	1^st^	1^st^
P60842	Eukaryotic initiation factor 4A-I	2^nd^	1^st^	1^st^
P04075	Fructose-bisphosphate aldolase A	2^nd^	1^st^	2^nd^
P52907	F-actin-capping protein subunit alpha-1	2^nd^	1^st^	1^st^
P04406	Glyceraldehyde-3-phosphate dehydrogenase	2^nd^	3^rd^	2^nd^
P07195	L-lactate dehydrogenase B chain	2^nd^	1^st^	2^nd^
P04083	Annexin A1	2^nd^	1^st^	2^nd^
P00338	L-lactate dehydrogenase A chain	2^nd^	1^st^	3^rd^
P62258*	**14-3-3 protein epsilon**	**2**^**nd**^	**1**^**st**^	**1**^**st**^
P26640	Valyl-tRNA synthetase	2^nd^	1^st^	2^nd^
P08670	Vimentin	2^nd^	1^st^	2^nd^
P35579	Myosin-9	2^nd^	1^st^	1^st^
P18206	Vinculin	2^nd^	3^rd^	2^nd^
P13639	Elongation factor 2	2^nd^	1^st^	1^st^
P07900	Heat shock protein HSP 90-alpha	2^nd^	1^st^	1^st^
P38646	Stress-70 protein, mitochondrial	2^nd^	1^st^	2^nd^
P10809	60 kDa heat shock protein, mitochondrial	2^nd^	1^st^	1^st^
P05787*	**Keratin, type II cytoskeletal 8**	**2**^**nd**^	**2**^**nd**^	**1**^**st**^
P06733	Alpha-enolase	2^nd^	3^rd^	1^st^
P26641	Elongation factor 1-gamma	2^nd^	2^nd^	1^st^
Q15029	small nuclear ribonucleoprotein component	3^rd^	1^st^	1^st^
P08195	4 F2 cell-surface antigen heavy chain	3^rd^	2^nd^	2^nd^
P53396	ATP-citrate synthase	3^rd^	1^st^	1^st^
O75369	Filamin-B	3^rd^	2^nd^	1^st^
P53992	Protein transport protein Sec24C	3^rd^	2^nd^	1^st^
P53618	Coatomer subunit beta	3^rd^	3^rd^	3^rd^
P14625	Endoplasmin	3^rd^	1^st^	1^st^
Q13263	Transcription intermediary factor 1-beta	3^rd^	2^nd^	1^st^
P02786	Transferrin receptor protein 1	3^rd^	3^rd^	1^st^
P55072	Transitional endoplasmic reticulum ATPase	3^rd^	1^st^	1^st^
O43390	Heterogeneous nuclear ribonucleoprotein R	3^rd^	2^nd^	3^rd^
P68371	Tubulin beta-2C chain	3^rd^	2^nd^	1^st^
P50454	Serpin H1	3^rd^	1^st^	1^st^
P00558	Phosphoglycerate kinase 1	3^rd^	1^st^	1^st^
P50395	Rab GDP dissociation inhibitor beta	3^rd^	1^st^	2^nd^
P40926	Malate dehydrogenase, mitochondrial	3^rd^	2^nd^	1^st^
Q99623	Prohibitin-2	3^rd^	2^nd^	3^rd^
P12429	Annexin A3	3^rd^	1^st^	3^rd^
P08758	Annexin A5	3^rd^	1^st^	3^rd^
O00299	Chloride intracellular channel protein 1	3^rd^	2^nd^	1^st^
P61289	Proteasome activator complex subunit 3	3^rd^	1^st^	1^st^
P09211	Glutathione S-transferase P	3^rd^	3^rd^	1^st^
Q06830	Peroxiredoxin-1	3^rd^	2^nd^	1^st^
P61026	Ras-related protein Rab-10	3^rd^	3^rd^	3^rd^
Q99714	3-hydroxyacyl-CoA dehydrogenase type-2	3^rd^	3^rd^	1^st^
Q08211	ATP-dependent RNA helicase A	3^rd^	1^st^	1^st^
Q14683	Structural maintenance of chromosomes protein 1A	3^rd^	1^st^	1^st^
P04264	Keratin, type II cytoskeletal 1	3^rd^	1^st^	1^st^
Q9NYL9	Tropomodulin-3	3^rd^	3^rd^	N/A
P21333	Filamin-A	N/A	3^rd^	3^rd^
P55786	Puromycin-sensitive aminopeptidase	N/A	2^nd^	1^st^
P52597	Heterogeneous nuclear ribonucleoprotein F	N/A	1^st^	1^st^
Q04695	Keratin, type I cytoskeletal 17	N/A	2^nd^	2^nd^
P42330	Aldo-keto reductase family 1 member C3	N/A	1^st^	3^rd^
Q08J23	tRNA (cytosine-5-)-methyltransferase NSUN2	N/A	2^nd^	N/A
P00966	Argininosuccinate synthase	N/A	1^st^	N/A
P10696	Alkaline phosphatase, placental-like	N/A	1^st^	N/A

Note: 1^st^ means that the protein was located as the 1^st^ level neighbors of RKIP; 2^nd^ means that the protein was located as the 2^nd^ level neighbors of RKIP; 3^rd^ means that the protein was located as the 3^rd^ level neighbors of RKIP; and N/A means no interaction of RKIP with those proteins or the level of their interaction was > the 3^rd^ level. *(bold font) means the validated proteins that interacted with RKIP.

In the protein network diagram that was derived from the database retrieval with the functional linage network, among the 72 RKIP-related proteins, 41 proteins were classified as the 1st level neighbors of RKIP, 21 proteins were classified as the 2nd level neighbors of RKIP, and 10 proteins were classified as the 3rd level neighbors of RKIP (Figure 5B, Table 2).

In the protein network diagram that was derived from the database retrieval with Predictome, among the 72 RKIP-related proteins, 43 proteins were classified as the 1st level neighbors of RKIP, 16 proteins were classified as the 2nd level neighbors of RKIP, 9 proteins were classified as the 3rd level neighbors of RKIP, and 4 proteins were found to not interact with RKIP (Figure 5C, Table 2).

Of the 72 RKIP-related proteins, only 35 proteins closely interacted with RKIP, as determined from the MiMI analysis. However, all 72 proteins had functional relationships with RKIP with the functional linage network and Predictome database analyses, and 69 proteins were predicted to closely interact with RKIP. In all three database analyses, 35 proteins were consistently found to be closely related to RKIP, including HSP90, 14-3-3ϵ, Keratin 8, IQGAP1, MYH9, CLH1, PLEC1, and EF2 (Table 2).

**Figure 6:**
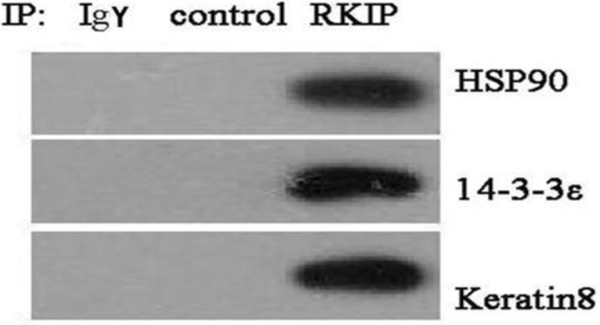
**The validation of RKIP-interaction proteins with immunoprecipitation and Western blot analysis.** The proteins were immunoprecipitated, with RKIP antibody, from the total protein extracts of SGC7901 cells and detected by Western blot analysis with antibodies against HSP90, 14-3-3ϵ, and keratin 8. Non-immune IgY antibodies were used to replace RKIP antibodies as negative controls (3×).

In our previous study, HSP90, 14-3-3ϵ, and keratin 8 were discovered to be significantly changed in the GC tissues compared with in the normal gastric mucosa tissues [3]. MiMI, functional linage network, and Predictome were three independent software and databases. Through the MiMI analysis, these proteins were classified as 2nd level neighbors of RKIP; through the functional linage network analysis, they were found to be directly functionally related to RKIP; and through the Predictome analysis, they were found to directly interact with RKIP. Those three proteins were further verified by Western blot analysis in combination with co-immunoprecipitation; the results confirmed the proteins’ interactions with RKIP. The results show that the protein expressions of the MS/MS-characterized HSP90, 14-3-3ϵ, and keratin 8 (Additional file 2: Table S2) were detected in the RKIP immune complex (Figure 6) and they were not detected when the RKIP antibody was replaced by IgY antibody.

## Discussion

The RKIP that was found to be down-regulated or absent in GC in our previous study [3], which is associated with the occurrence, differentiation, invasion, and metastasis of GC. Recent studies further found that RKIP can prevent the phosphorylation and activation of MEK that is mediated by the RAF-1 and MAPK signaling pathways [9]. RKIP can also participate in the regulation and control of the G protein coupling receptor signaling pathway and the NF-kB signaling pathway [16,27]. These signaling pathways play important roles in cell growth, proliferation, differentiation, and tumorigenesis processes, which strongly suggests that abnormal expression of RKIP is involved in tumorigenesis. Our previous study also revealed that the abnormal expression of RKIP plays an important role in the growth and differentiation process of GC.

Among numerous signaling pathways, the MAPK pathway plays a crucial role in inflammatory signal transduction, apoptosis, and tumor cell proliferation, and it controls a variety of internal metabolic process. Extracellular signal-regulated kinase (ERK) is a member of the MAPK family. Its RAS/RAF/MEK/ERK signal transmission pathway is the core of the signal network, which is involved in the regulation of cell growth, development, and division [9,17,28].

RKIP can prevent the phosphorylation and activation of MEK that is mediated by RAF-1; thus RKIP can also influence the MAPK signaling pathway by combining with its interacting proteins. Therefore, we aim to study the RKIP-interacting proteins in GC and the action mechanism of the RAF/MEK/ERK signaling pathways which are influenced by RKIP in this paper.

Proteomics in combination with fusion protein expression is the first approach to characterize RKIP-interacting proteins. A total of 72 RKIP-related proteins were identified in the human gastric carcinoma cell line SGC7901. The identified proteins belong to different functional categories, including those of metabolic enzymes, molecular chaperones, biological oxidation-related proteins, signal transduction-related proteins, cytoskeleton-related proteins, protease-related proteins, and others. Among those 72 proteins, only 35 proteins were found through the MiMI analysis to have existing interactions with RKIP; however, through the functional linage network and the Predictome database analyses, each of the 72 proteins was found to be functionally related to RKIP, and 69 of the proteins were found to closely interact with RKIP.

Studies have demonstrated that some of the 72 related proteins, including MYH9, IQGAP1, annexin A1, vimentin, and GSTP1, might play an important role in the occurrence, differentiation, invasion, and metastasis of GC. The protein MYH9 functions in cytokinesis, cell motility, and the maintenance of cell shape. Many studies suggest that MYH9/NMHC-IIA plays a key role in tumor cell invasive behavior; and a recent study shows that the inhibition of MYH9/NMHC-IIA expression can inhibit the metastasis of GC cells [29]. IQGAP1 was discovered to be upregulated in GC, and its absence corresponds to a good clinical prognosis [30]. The loss of annexin A1 expression has been significantly associated with advanced stage lymph node metastasis, an advanced disease stage, and poor histological differentiation. The ANXA1 expression decreased significantly as GC progressed and metastasized; this result suggests the importance of ANXA1 as a negative biomarker for GC development and progression [31]. Studies have demonstrated that the expression of vimentin was significantly upregulated in GC tissue and that the elevated vimentin expression was strongly correlated with lymph node metastasis, lymphatic invasion, perineural invasion, and pathological staging [32]. A recent study found that GSTP1 mRNA and protein were present in drug resistant gastric cells (including SGC7901 cells) and that the down-regulated expression of GSTP1 was related to somatic promoter hypermethylation and impaired ERK signaling in GC cell lines [33].

HSP90 and 14-3-3 were found to be significantly changed in the GC tissues compared with in the normal gastric mucosa tissues in our previous study [3]. In this study, HSP90 and 14-3-3 were identified as 2nd level neighbors of RKIP by the MiMI analysis (Figure 5A), as directly functionally related to RKIP by the functional linage network analysis (Figure 5B), and as likely to directly interact with RKIP by the Predictome analysis (Figure 5C). The interactions of RKIP with HSP90 and 14-3-3 proteins were verified by Western blot analysis in combination with co-immunoprecipitation (Figure 6). HSP90 and 14-3-3 proteins also play an important role in the RAF/MEK/ERK signaling pathways [34,35].

The heat shock protein 90 family is a group of highly conserved proteins and expressed in all eukaryotic cells. HSP90 is over-expressed in a variety of tumor cells. Liu et al. have confirmed that HSP90 is also over-expressed in SGC7901 GC cells [36]. In recent years, studies have confirmed that heat shock protein family members such as HSP90 may directly affect the anti-apoptotic mechanisms of cells, and that, because of HSP specific functions, HSP90 plays an important role in the regulation of the cell’s anti-apoptotic reaction. In the RAF/MEK/ERK signaling pathways, the vital RAF-1 and MEK kinases have been found to be substrate proteins of HSP90. Experiments show that HSP90 is important for the regulation of RAF-1 protein activity, intracellular positioning, and stability. The use of HSP90 blockers to prevent the interaction of HSP90 and RAF-1 can lead to a quick decline of intracellular RAF-1 protein, block downstream MAPK activation, and induce apoptosis [34]. As the previous studies have proven, HSP90 protein plays an important role in the activation of the RAF/MEK/ERK signaling pathways.

The 14-3-3 signaling proteins are a group of multifunctional eukaryotic proteins that are highly conserved and widely distributed. Some subtypes of 14-3-3 were found in GC cell lines with high expression [37]. The 14-3-3 protein, in partnership with cancer gene or proto-oncogene products, participates in cellular signal transduction, plays a role in RAF/RAS/MAPK signaling, directly affects the signal transmission related to phospholipid activity and Ca^2+^, and is involved in T cell activation and cell apoptosis [10,38]. Studies have confirmed that in the RAS/RAF/ERK/MEK signal transduction pathways, the combination of 14-3-3 and RAF multiple sites were the collaborative factor of RAF-1 and RAS. Additionally, RAF-1 was transferred to the cell membrane by 14-3-3, fixed in cell membrane by RAS, and then activated by 14-3-3. Therefore, 14-3-3 is the enzyme downstream of RAS, in the MAPK pathway, with which RAF-1 interacts [39,40]. Protein 14-3-3 plays a major role in maintaining the stability of the RAS/RAF-1 complex and in forming membrane sites. The involvement of 14-3-3 protein in the regulation of the RAF/MEK/ERK signaling pathways has been reported [35]. Current research suggests that 14-3-3σ, a member of the 14-3-3 protein family, has tumor suppressor activity, while other members of the 14-3-3 protein family have tumor promotion activity. In our initial proteomic studies, the 14-3-3 protein that was found to be an RKIP-interacting protein was 14-3-3ϵ. The overexpression of 14-3-3ϵ in vitro can limitedly prompt the abnormal growth of renal tumor cells [41]; and the cleavage and translocation of 14-3-3 epsilon is significantly associated with the inhibition of colon cancer cell proliferation [42]. Further study on the function of 14-3-3ϵ in the tumor signaling pathway would be warranted.

### Specificity of identified RKIP-interacting proteins

Specificity is a key issue in this study. In order to rule out the nonspecific bound proteins, three experiment groups were designed as pcDNA3.1-RKIP-3xFLAG, pcDNA3.1-3xFLAG (blank-carrier) control group, and the blank control group. The targets in this study are RKIP and its interacting proteins. The blank-carrier control pcDNA3.1-3xFLAG without RKIP fusion is a good, effective, and comparable control because the relative literature [18-26] has been reported that 3xFLAG coupled its specifically effective antibodies is an efficient and high-performance system to purify flag-tagged protein and its interacting partners. In addition, the small size of the FLAG peptide tag is not likely to obscure other epitopes, domains, or alter function, secretion, or transport of the fusion protein, and affect the interaction of the fusion protein with its partners [18]. Meanwhile, the previous quantitative proteomics studies discovered that both of 14-3-3 protein and RKIP were differentially expressed proteins in gastric carcinoma tissues [3]; however, this 3XFLAG peptide tag experiment confirmed the interaction of RKIP with 14-3-3 protein.

## Conclusions

In summary, the targeted RKIP-interacting proteins were analyzed with proteomic methods to address the molecular mechanisms and biological roles of RKIP in GC. A total of 72 RKIP-related proteins were identified. All of these 72 proteins were directly or indirectly related to RKIP. Three RKIP-interaction protein network diagrams were constructed with MiMI, the functional linage network, and Predictome to address the molecular pathways of the functional activities of RKIP. The interactions of RKIP with HSP90, 14-3-3 protein, and keratin were verified by co-immunoprecipitation and Western blot analyses. Studies on HSP90 and 14-3-3 proteins in the RAS/RAF/MEK/ERK signaling pathways have been reported [34,35]. Those two proteins (HSP90 and 14-3-3ϵ) have close relationships with this signal pathway and with the transferring and activation of RAF-1. HSP90 and 14-3-3ϵ were also associated with the growth and differentiation process of GC. Because these two specific proteins are involved in the mechanisms by which RKIP inhibits GC development, HSP90 and 14-3-3ϵ proteins can consider as early-stage biomarkers and targets for therapeutic strategies to treat GC.

## Competing interests

The authors declare that they have no competing interests.

## Authors’ contributions

HG conceived, designed and carried out the work that led to the submission, and played an important role in interpreting the results. XZ was involved in drafting the manuscript and revising it critically for important intellectual content,and also played an important role in interpreting the results, approving the final version and corresponding. GZ participated in its design, coordination and corresponding and approved the final version. LY carried out the data collection and participated in the analysis of mass spectrometry. WCSC participated in revising it critically for important intellectual content. ML carried out the MS/MS-identification of proteins. TL participated in the purification of RKIP fusion proteins. ZC participated in its design and proteomics data explanation. All authors read and approved the final manuscript.

## Pre-publication history

The pre-publication history for this paper can be accessed here:

http://www.biomedcentral.com/1471-2407/13/536/prepub

## Supplementary Material

Additional file 1: Table S1 MS/MS identification of proteins that interact with RKIP. Click here for file

Additional file 2: Table S2 MS/MS spectra of the corresponding peptides identifying three validated RKIP-interacting proteins. Click here for file
